# Physical activity in infancy and early childhood: a narrative review of interventions for prevention of obesity and associated health outcomes

**DOI:** 10.3389/fendo.2023.1155925

**Published:** 2023-05-24

**Authors:** Natalie Eichner-Seitz, Russell R. Pate, Ian M. Paul

**Affiliations:** ^1^ Department of Pediatrics, Penn State College of Medicine, Hershey, PA, United States; ^2^ Department of Exercise Science, Arnold School of Public Health, University of South Carolina, Columbia, SC, United States

**Keywords:** obesity, exercise, pediatric, body composition, motor development, infant, toddler, preschool

## Abstract

In the context of the childhood obesity epidemic, this narrative review aims to explore opportunities to promote physical activity (PA) between birth and age 5 years as well as the health outcomes associated with PA in early childhood. Although early childhood is an ideal time to promote healthy habits, guidelines for PA have often ignored early childhood given the limited evidence for children <5 years old. Herein we discuss and highlight infant, toddler and preschool age interventions to promote PA and prevent obesity both in the short and long-term. We describe novel and modified interventions to promote improved early childhood health outcomes, encompassing cardiorespiratory, muscle, and bone strengthening components necessary for short-term motor development and long-term health. We call for new research aimed at developing and testing innovative early childhood interventions that may be performed in home or childcare settings, monitored by parents or caregivers.

## 1 Introduction

The high prevalence of obesity in all age groups is a major public health concern. Alarmingly, recent worldwide data suggests that an estimated 39 million children under the age of 5 were classified as having obesity in 2020 and that number is expected to rise to 40 million by 2030 (WHO 2021). Primary prevention is likely the best solution, as recent simulation data in the *New England Journal of Medicine* suggest that “a 2-year-old who is obese is more likely to be obese at 35 years of age than an overweight 19-year-old.” (1)

Physical activity (PA), a multi-faceted behavior typically associated with increased energy expenditure and load-bearing forces, has long been endorsed as a means of health promotion and disease prevention across the lifecourse (2, 3), although comparatively, very little work has conducted interventions in the first few years after birth. Infancy (0-12 months), toddlerhood (12-36 months) and the preschool period (36-60 months) are unique periods of robust developmental plasticity where health behaviors such as PA can have lasting metabolic and behavioral consequences. Obesity-prevention interventions to increase PA during this time period promote improvements in health outcomes, including weight, that persist into childhood (4–6) and early adulthood years (7). Despite these promising results, there are few specific recommendations for PA in infants (8–10) and toddlers (9, 10) with slightly more recommendations for preschoolers (10, 11). In 2011 the Institute of Medicine acknowledged the dearth of research and lack of published consensus on recommendations related to infant PA (12). Although in more recent years, several countries and the World Health Organization (WHO) have published 24-hr movement guidelines for children under 5 years old that include specific recommendations for time spent in active play, specific to each year (13–15).

The challenges related to increasing PA in early childhood, which we define as birth to age 5 years, are unique and dependent upon child development within that period. To our knowledge, no review of early childhood PA interventions has considered these unique difficulties in conjunction with proposed novel solutions that may work to address these challenges. Therefore, with this review, we aim to 1) summarize evidence and identify gaps related to PA based on the developmental timeline of infancy, toddler and preschool; 2) highlight the impact of interventions on health outcomes (such as body composition and bone health), and 3) propose novel PA interventions specifically designed to address these identified gaps and improve health outcomes.

## 2 Methods

### 2.1 Data sources and search strategy

This was designed as a narrative review, to include a search of the following electronic databases through January 2023: EBSCO (CINAHL); Cochrane Library (Central) and OVID (EMBASE, MEDLINE, PsycINFO) and Web of Science (all). Key term used in the search included *infant, toddler, early obesity; physical activity; exercise*, floor play, *bone health, body composition, adiposity.* Reference bibliographies were also searched to identify relevant studies as were additional publications or reviewers identified as relevant by study authors.

### 2.2 Study selection and data extraction

Studies were included if the following criteria were met: 1) reported results from a longitudinal, experimental, or cross-sectional study, 2) reported any movement behavior to include physical activity or exercise (acute or chronic interventions of any intensity or duration, supervised or unsupervised). Studies were considered eligible if published in English included participants with or without obesity aged 0-5. Studies were excluded if only animal data was reported. Study design, sample size, publication year, age of participants, type of participants, description of PA or exercise intervention, and associated health outcomes were extracted from each identified publication.

## 3 PA interventions in infancy (0-12 months)

Within the last decade there have been promising trials done to promote healthy behaviors during infancy (5, 16, 17) that may extend into early childhood (18) (19). However, since the recommendation by the American Academy of Pediatrics (AAP) to include tummy time as part of an infant’s routine in the 1990s, there have been very few, if any, novel recommendations for providers to promote PA prior to age 1 year (20). This is unfortunate, as pediatricians can be effective promotors of PA to their patients (21) with frequent points of contact during the first several years (22). Recognizing this unique opportunity, a newly published report from the AAP encourages pediatricians to provide a clearly documented PA “prescription” that will allow for other providers, parents, and caregivers to “administer” an accurate dose of PA (20). As with any successful medication prescription and regimen, consideration must be given to timing, dose, and even individual factors such as adherence. Unfortunately, very little physiologically based research exists today that can inform PA prescriptions for infants.

Little is known regarding the exact mechanisms by which infant motor development, PA, and rapid weight gain early in life are related. Some speculate that it relates to critical periods of infant development where rapid cell development and growth occur, leaving infants vulnerable for increased risk of inappropriate weight gain (23–25). Number of adipocytes, a major predictor of fat mass in adulthood, is determined early in life (26) potentially explaining the positive association between rapid infant weight gain and persistence of obesity into both childhood and adulthood (25). Increased infant weight gain has also been shown to promote epigenetic modifications that regulate gene expression associated with persistent weight gain into childhood (27).

### 3.1 Tummy time

**Figure 1:**
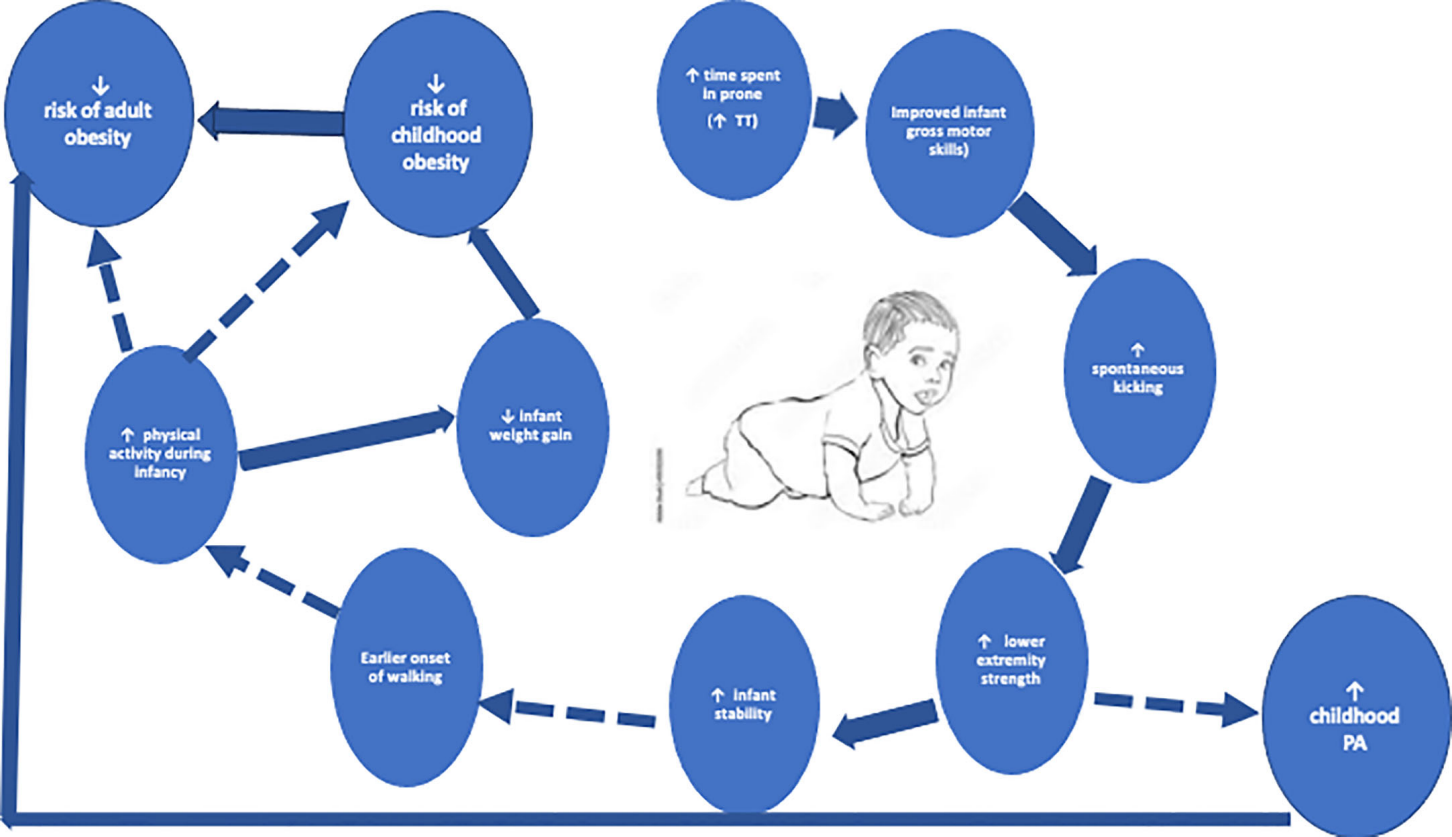
The reinforcing loop of increased tummy time, physical activity, and decreased obesity. Solid lines are supported references within the text. Dashed lines are opportunities for investigation.

Infant positioning is one of the earliest activities to promote PA. Beginning on a child’s first day home from the hospital, the AAP recommends short periods of awake time in the prone position, playing and interacting with their environment in a meaningful way. Time should slowly be increased as the child begins to exhibit activity enjoyment with the end goal of accumulating 30 minutes daily. Known as “tummy time” (28) this recommendation has remained constant as one of the few recommendations for promoting infant PA (29). Tummy time is supported by several studies showing delayed gross motor development in infants who did not meet the recommended time spent in prone position (30, 31). This delay in motor development may contribute to accelerated infant weight gain by limiting the amount of PA (**Figure 1**) (32, 33). A recent systematic review found that tummy time was inversely related to BMI z-score in the first year, and positively related to gross and total motor development, as well as the ability to move while prone, supine or crawling (29). Additional data have shown that increased unrestricted movement during infancy has been associated with decreased waist circumference z-score and improved weight‐for‐length *z-*scores between ages 9 and 24 months (34). Most of the reports regarding tummy time and unrestricted movement are observational and lack objectively measured data with tools such as accelerometers (29). However, recent promising data has shown that higher levels of infant PA measured by ankle-worn accelerometers are associated with lower central adiposity (35). Data are lacking regarding associations between tummy time and infant unrestricted movement with other outcomes such as muscle strength and bone health (29, 31, 36).

### 3.2 Environmental and parental interventions

Targeting parenting practices through educational programs is one of the most well-documented interventions to assess both short- and long-term impact on infant PA (4, 6, 16, 18, 37). Interventions to date typically delivered for the purposes of obesity prevention have varied in focus and depth, with topics ranging from responsive parenting techniques (5), appropriate breastfeeding and sleep habits (6) as well as anticipatory guidance on infant diet and PA (16). The findings have been mixed in terms of impact on weight-related outcomes though none were designed to directly assess impact on PA as their primary outcome nor was PA during infancy objectively measured.

### 3.3 Treadmill interventions

Novel treadmill-based interventions have been studied in children with developmental disabilities to improve attainment of neuromotor skills that might otherwise be delayed (38). The utilization of partial body weight supported treadmill training in infants as young as 4 months decreases the delay in onset of walking, and improves walking gait in infants with and without a disability (38–40). These studies have found no impairment of such interventions on child growth or development. The authors speculate that increasing the onset of walking, in conjunction with improved stability during locomotion, promotes increased PA throughout infancy (41). Given the research highlighting the association between motor development in childhood and PA engagement in adolescence (42, 43), these patterns may promote PA engagement across the life course. To date, however, no extensive work has been done utilizing treadmill interventions in normally developing infants as young as 4 months. Ulrich et al. (39) highlight that these interventions can be done safely while positively impacting infant motor and muscle development.

### 3.4 Muscle and motor development

No recommendations exist for “strength training” i.e., muscle and motor development, in infancy. Although the idea of “strength training” during infancy may seem premature, appropriate skeletal muscle development during this time can have long-term consequences on body composition (44) and willingness to engage in activity throughout the lifespan (45), key mediators of body composition in adulthood. Genetics do play a major predeterminant of muscle fiber composition (46), however, environmental factors likely play an important role given well-known trials highlighting “fiber reprogramming” in response to exercise training (47). Interestingly, some report increases in fibers related to enhanced fat metabolism in infants (Type 1 and Type 2a) vs. increases in fibers related to impaired fat metabolism (Type 2b) in adults (48). This holds clinical relevance as type 2b fibers have been associated with obesity and insulin resistance in adolescents and adults (48). It is unknown if these fibers can be maintained or reprogrammed in infancy to promote an improved metabolic profile later in life. Animal models have shown significant increases in Type 1 and Type 2a fibers following a unilateral leg kicking program (49). Implementation of a similar program in infancy might promote a favorable muscle fiber profile, mitigating increases in Type 2b fiber types related to obesity and insulin resistance later in adolescence and adulthood.

The above intervention is relevant, as spontaneous, rhythmical kicking in infancy typically seen around 1 month (50) can promote strength improvements through flexion and extension of the knee joint, presenting a unique way to improve strength early in development. As a certain amount of strength in the lower extremities is necessary for walking skills, such as pull-to-stand, promoting kicking during infancy can lead to earlier onset of walking in children (51). This can become a self-reinforcing loop, with those children walking earlier becoming more stable and subsequently engaging in more PA throughout infancy and childhood **(** **Figure 1**). Very recent work in infants 6-7 months highlights this relationship in a multivariate analysis of infant PA counts with more advanced motor development (52)

Very little work has assessed interventions designed to promote increased strength or muscle development in infants, though there have been efforts to promote proper neurodevelopment in children born prematurely using sensorized toys (53). These toys were designed to encourage spontaneous behaviors such as reaching, kicking, and grasping, thereby promoting muscle and strength development. Like treadmill interventions described above, these tools were primarily developed for at-risk preterm, low birth weight infants to improve motor skills and improve body composition development (53–55). However, the known overlap between motor development and improved weight status in youth (56, 57) **(** **Figure 1** **)** highlights the utility of such interventions in high birthweight infants, another “at-risk” group. Given the toy’s unique ability to measure forces generated by the infant (54), this would provide novel quantitative insight into tracking and development of anaerobic power and muscular strength during infancy, with the goal of better understanding the role “strength training” might play in mitigating weight gain throughout the lifespan.

### 3.5 Bone health

As with other metabolic and body composition outcomes, bone mass accrual during infancy is accelerated and occurs at one of the most rapid rates an individual will experience during their lifetime. Bone mineral content and density is increased in response to repetitive and varying load-bearing activities *via* increased force and strain. Unfortunately, despite this period of significant potential for bone accrual, many infants do not engage in load-bearing activities that would elicit the necessary stimulus to prompt remodeling changes in the bone. Two small studies have attempted to determine if increased PA during infancy affects bone mineral density showing mixed results (58, 59).

### 3.6 Current limitations in assessment of PA in infancy

There is significant variability in infant gross motor development limiting the reliability of PA measurement, especially within the first 3 months after birth (60). Additionally, there is a wide range of approaches for PA measurement, ranging from physiologic to behavioral (61). Direct accurate measurements of energy expenditure in infants using methods such as indirect calorimetry (62) or doubly labeled water (63) can be done, although lack of accessibility and increased cost limits its utilization in large population studies. Other more readily available means of assessment include questionnaires, observation, and accelerometers. The limitations of questionnaire and observation are well-known and documented elsewhere (64) with major drawbacks including lack of quantifiable data that directly measures PA. Accelerometers are the “gold standard” of PA assessment in infancy (65) however, this too presents unique age-specific confounding factors including the influence of caregiver lifting and carrying of the infant, which some have shown accounts for up to 40-50% of measured activities in infants wearing accelerometer (66). The best method for monitoring PA in children younger than age 2 years warrants more extensive testing, to further our understanding of PA patterns in infancy. Fortunately, current research by Pate et al. is being done to investigate this question (67).

## 4 PA interventions during the toddler years (12-36 months)

**Figure 2:**
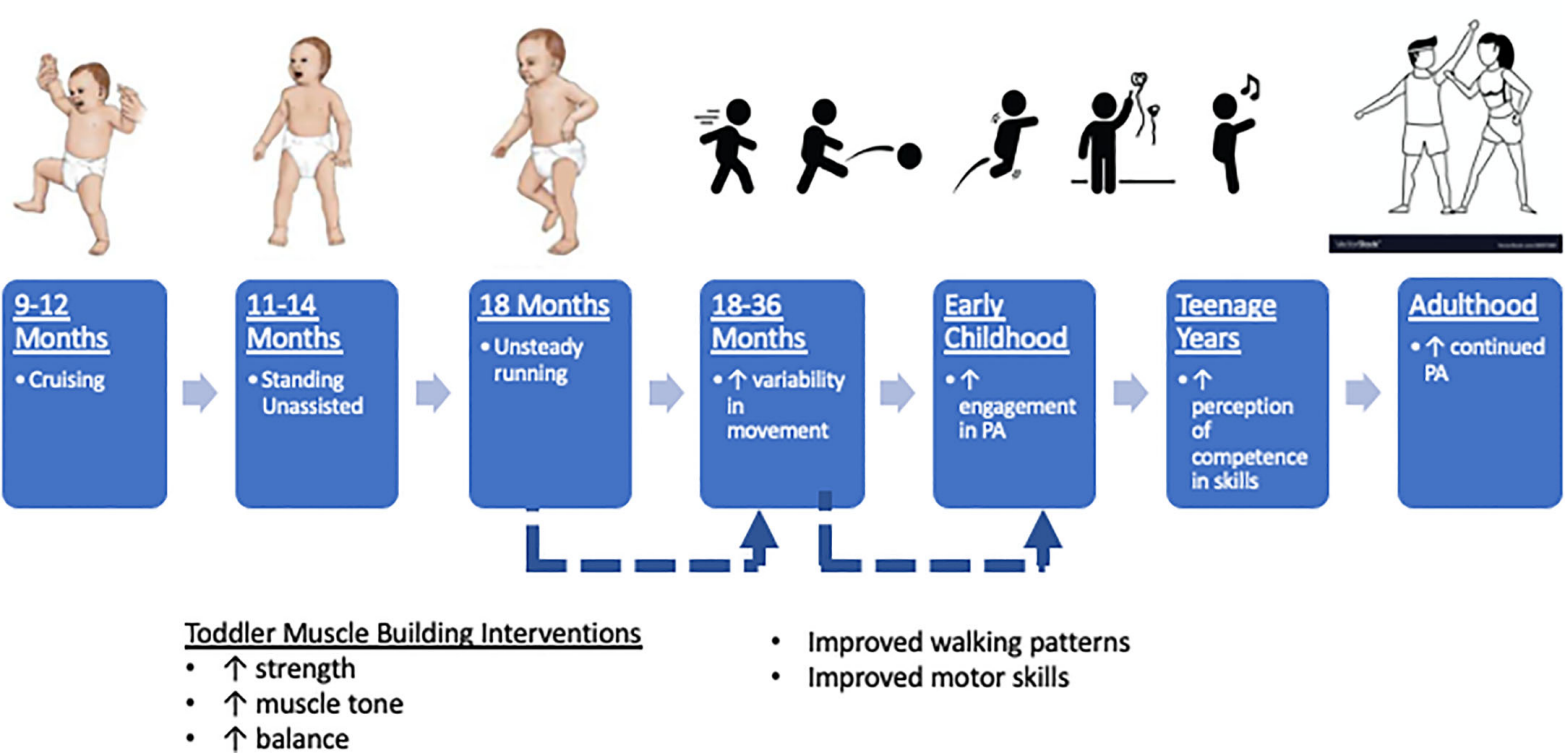
Toddler strength training interventions to promote continued engagement of physical activity into adulthood. Solid lines are supported references within the text. Dashed lines are opportunities for investigation.

By the end of their first year, most infants have developed the motor patterns necessary for locomotion, such as cruising (7-11 months) and standing unassisted (11-14 months), although they typically appear to be unsteady (hence the name “toddler”). Beyond 18 months, the toddler gait has developed sufficiently that fast walking closely resembles running. This increased freedom in both motor skill and function promotes variability in PA options compared to infants. The impact of PA in toddlerhood versus infancy may also have differential effects. PA in toddlerhood is associated with greater improvements in bone and skeletal health (68) as opposed to improved adiposity and motor skills in infancy (69). **(** **Figure 2** **)** These findings highlight that context of PA is especially important when considering interventions during the toddler years, as this age group’s activity patterns tend to be more sporadic, involving short, rapid bursts of movement (70).

### 4.1 Environmental and parental interventions during toddlerhood

Nearly 40% of US toddlers are cared for exclusively by a parent in the home (71). Therefore, home-based trials provide the opportunity to engage both child and caregiver in easily accessible ways. Mother-toddler diet and PA behaviors are significantly related (72, 73) since toddlers learn through modeled behaviors (74). Targeted maternal lifestyle interventions significantly increase toddler PA, although this may not influence toddler weight gain (75).

Childcare centers serve as another means of administering PA interventions. Extensive research has been done (76) in these settings to estimate not only the amount of PA toddlers engage in throughout the day, but also, the intensity levels of this PA, stratifying data based on light to moderate-to-vigorous physical activity (MVPA). A recent meta-analysis highlights that although toddlers tend to engage in >180 minutes of PA/day (exceeding the recommendation by the WHO), very little of this time is spent in activities quantified by moderate or high intensity, which may have long-term health consequences (69). In response to this concern, some have advocated for modifying the center environment with portable play equipment (77) or increased scheduled time spent in a non-restrictive outdoor environment (78) as nearly 15% more MVPA has been reported outdoors than indoors in childcare settings (79).

Other interventions to promote PA in toddlers enrolled in childcare settings include modification of center policies to promote increased active play with verbal reminders from staff trained in PA (80, 81) and limited screen time during recreational periods (82). Unfortunately, most centers in the US do not observe recommendations for PA promotion; nearly 90% of childcare services provide less than two hours of active play per day (83). With these considerations in mind, multi-component interventions addressing both childcare center practice and policies have been developed and implemented with modest results (76). Most importantly, these trials did not assess the direct impact of the intervention on PA in the cohorts of toddlers. Interventions to directly increase toddler PA across settings lacks specific and targeted goals informed by previous research; effective interventions most likely target multiple components (i.e. environment, caregiver etc.)

### 4.2 Muscle and motor development

Similar to interventions in infancy, most strength programs done in toddlerhood to promote muscle building have been in toddlers with either intellectual or physical disabilities (84–86). Although a recent systematic review by Pate et al. noted a significant relationship between higher levels of physical activity with bone health in healthy children (87). In a very small cohort of toddlers with Down Syndrome (n=5), Sayers et al. found that an individualized, sequential at-home 8-week pediatric strength intervention designed to improve strength, tone and balance saw improved walking patterns (84). This program was designed using the theoretical basis of progressive and interactive facilitation, which combines both proprioceptive stimuli and neurodevelopmental patterning delivered through exercise. Initially, there were significant concerns that parents would not feel confident in administering this type of intervention in the home. This study provided proof-of-concept, as parents who were trained as part of this program believed they could successfully implement this intervention. Parents also believed that the intervention was effective and efficient for improving their child’s motor control (88). To our knowledge, no current interventions have implemented a theoretically- based pediatric strength intervention for children without disabilities.

Swimming, or aquatic therapy, is another full-body aerobic and motor-development intervention utilized as a conjunctive therapy for children with developmental delays or disabilities. The benefits of swimming have been well-documented in these children and include improved strength, increased active and passive range of motion, improved postural control and increased quality of life (89–91). Swimming also serves a practical purpose as the American Academy of Pediatrics recommends that all children 1 year of age or older learn swimming to prevent drowning (92). Additionally, the buoyancy of water provides a unique environment through which the toddler can develop postural control and engage in partial or full range of motion activities utilizing multiple large muscle, promoting increases in strength and motor development. Once again, despite effective swimming trials being conducted in toddlers with disabilities, no studies have been done in toddlers without a disability to determine if motor strength, range of motion or motor control would be improved similarly. This is relevant, as improvements in motor control during these early years could have long-term implications for motor development beyond childhood. A small study (n=19) showed that “baby swimming” improves hand-eye coordination and balance at a 4-year follow-up compared to children (93) (94). Some speculate that enhancements in motor development during this early period not only promote better motor skills, but also increase the child’s perception of their sports ability, reinforcing their continued engagement in PA throughout their life ***(*** 94 ***).***

### 4.3 Cardiometabolic and bone health

High intensity interval training (HIIT) interventions might be used to promote both cardiometabolic and bone health in toddlers. HIIT consists of short bursts of vigorous activity followed by recovery bouts of moderate activity and has been used to encourage individuals (primarily adults) to gain the benefits of PA when perceived lack of time is a barrier. Compared to traditional exercise training, HIIT has been shown to significantly improve cardiometabolic outcomes such as systolic blood pressure and VO2 max (a measure of cardiorespiratory fitness) in youth with obesity (95). Importantly, children perceive it to be more enjoyable (95). Given that the pattern of HIIT is very similar to how toddlers engage in active play, interventions utilizing this program remain highly applicable in this population. Unfortunately, recommendations for incorporation of HIIT into toddler programs remain vague due to the lack of current literature and methodological limitations of the data that does exist (96).

In addition to improving cardiometabolic outcomes (95) incorporation of HIIT interventions into toddler research may also improve skeletal health. Bursts of bone-loading activity with only short bouts of recovery accumulating in 2-3 minutes is a potent stimulus that has significant osteogenic effects (97). These benefits may occur in a dose-response, as MVPA has strong associations with improved bone and skeletal health in toddlers, with higher intensity of PA associated with even greater improvements in these outcomes (68). Children who engaged in higher amounts of MVPA between 2-3 years had increased bone mineral content and density at 5 years (18). Although not explicitly assessed, these interventions most likely also targeted muscle development and strength, as growing muscle is an essential component of increasing the load on a child’s growing bone, known as the muscle-bone unit. Given this relationship, we hypothesize that HIIT interventions in this cohort would also positively influence muscle development, in addition to cardiometabolic and bone health, leading to improved weight outcomes.

### 4.4 Current limitations in assessment of PA in toddlerhood

As highlighted above, the current research assessing strength and motor development programs has consisted of small patient cohorts or specific patient populations, such as children with disabilities, limiting interpretation of the data available. The new AAP recommendations for swimming classes for those ages 1-4 years offers an opportunity for research on health benefits of swimming for toddlers (92). As with the treadmill interventions for infants, findings among children with disabilities provides proof of concept that these trials can be implemented safely and effectively when done under proper supervision.

## 5 PA interventions during the preschool years (36-60 months)

**Figure 3:**
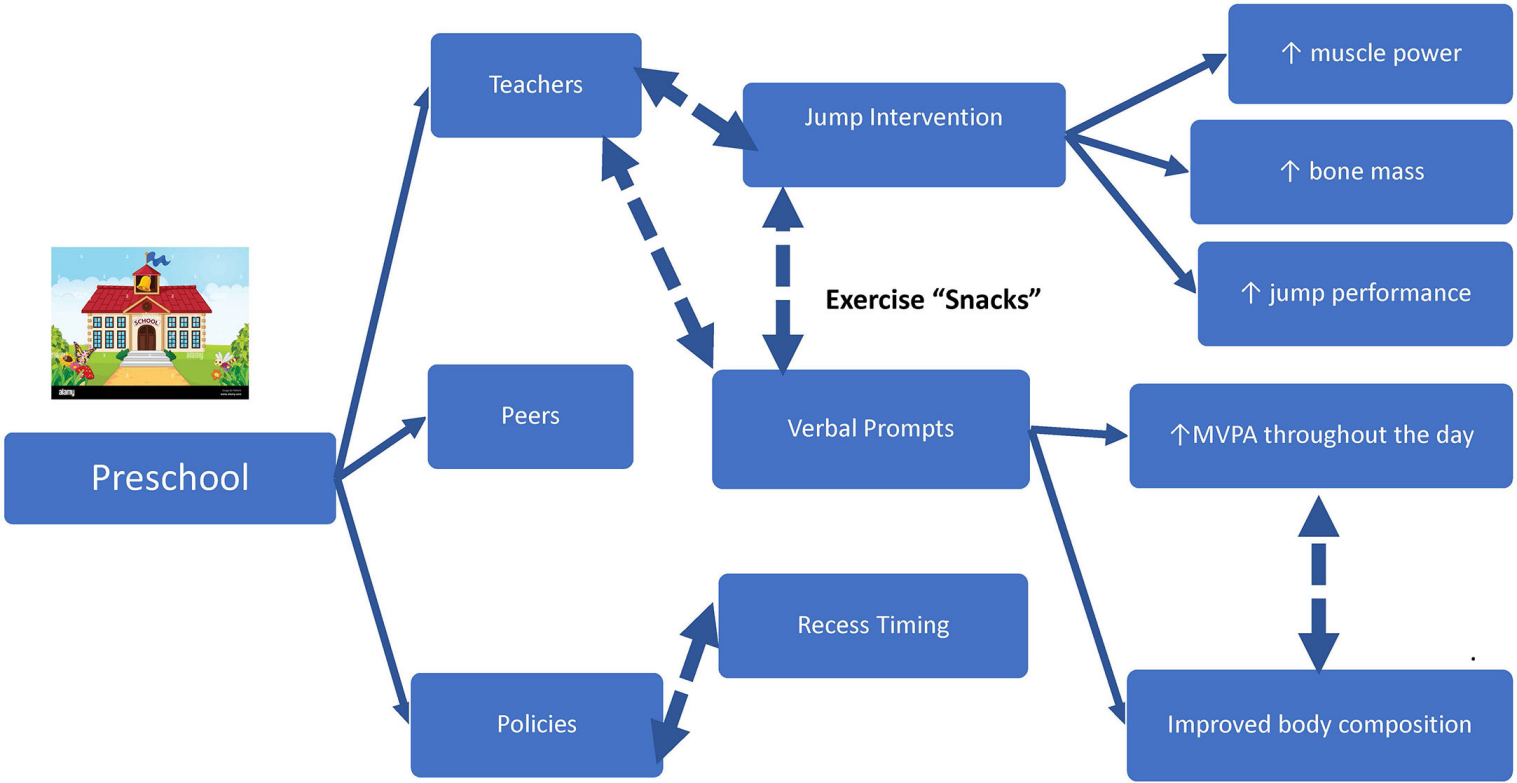
Exercise snacks to promote physical activity in preschool. Solid lines are supported references within the text. Dashed lines are opportunities for investigation.

In comparison to infancy and toddlerhood, more recommendations exist for preschool children aged 36-60 months such as a goal of 3 hours of PA/day (9–11, 13) though less than half of children meet those recommendations (98). More specific recommendations provided by the CDC encourage active play “everyday throughout the day” that should include aerobic, muscle-strengthening, and bone-strengthening activities (11). These guidelines are much more specific in comparison to the recommendations currently made in infancy and toddlers, so as expected, more trials have been completed (81, 99–101). Despite the number of interventions that have been done to increase children’s PA, findings have been inconsistent and typically lack objective measures of PA (79, 98, 101–103) which may, in part, explain discrepancies amongst the data (**Figure 3**).

### 5.1 Environmental and parental interventions

Like toddlers, overweight preschoolers are approximately three to six times more likely to have at least one parent with obesity in comparison to children who are not overweight (101). Interestingly, Trost et al. reported no differences between these two groups in factors previously thought to mitigate risk for obesity, such as parental modeling of PA, parental support for PA, or the number of toys/equipment available at home for active play (104). Other PA factors in this age group to consider include personality (101) and peer group (105).

As more than half of preschool aged children in the US attend childcare (106), interventions conducted in preschool or childcare settings provide access to a large cohort of children who engage in mostly sedentary behaviors during that time (65, 107). Unfortunately, children’s PA levels are highly variable among preschools, suggesting that policies and practices significantly influence the day-to-day behaviors of its students (108). As in toddlers (74, 76, 77, 80), strategic modifications of the school environment on the playground can increase PA (102). More specifically, preschoolers are more active during the first minutes of recess when compared with the minutes prior to reconvening to go back inside (100) highlighting the strength of incorporation of multiple, shorter periods of recess as opposed to one long recess bout (109).

Given that increasing the amount of time spent in PA in preschool programs can be challenging and has had little success in the past, interventions should consider focusing on the intensity of activity in this age cohort. Preschool teachers can be trained to promote MVPA throughout the day (110, 111) which can have a meaningful impact. Higher levels of daily MVPA correlates with improved body weight in a large cohort of preschool children (112). Given that higher levels of MVPA are typically associated with higher levels of overall childhood PA ***(*** **Figure 2** ***)*** and that higher levels of childhood PA are associated with improved body composition in adulthood ***(*** **Figure 1** ***)***, the long-term effects of increasing intensity of exercise during the early childhood years must be highlighted in future trials.

### 5.2 Muscle and motor development

For many years, there have been extensive debates surrounding the safety and feasibility of initiating “strength training” in youth (i.e. muscle building activities) (113). When done correctly under the supervision of appropriately trained personnel, such interventions can be safely administered (114). Very recently, a 10-week exercise program administered 3x/week in preschool and kindergarten classrooms significantly increased jump performance and muscle power (115). The program consisted of progressive repetitions of musculoskeletal loading activities such as lunges, ankle hops, lateral jumps, squats, and other exercises that could easily be done in a classroom or daycare setting. The proven feasibility of this intervention is timely, as there have been health benefits of brief “exercise snacks” in other age groups consisting of scheduled, short bouts (15-30 seconds) of vigorous activity (e.g. stair climbing) designed to break up prolonged periods of sedentary time. These snacks improve cardiometabolic outcomes such as insulin sensitivity in adults with obesity (116), though effects of “exercise snacks” in youth are unknown. However, these findings highlight the potential impact a time-efficient and easily administered novel intervention can have in childcare settings particularly those that promote anaerobic power and strength in the early childhood years, given the many known health benefits independent of aerobic fitness (117). These interventions must be easily implemented either at home or in a daycare or school setting, while not being overly complicated or increasing the amount of burden on caregivers or teachers.

One novel intervention we propose is the incorporation of the “exercise snacks” (116) described above with the muscle-building activities described by Wick et al. (115) but modified to be a very brief bout of activity. This requires little time, basic training, and could easily be incorporated into busy childcare and classroom settings with meaningful impact. The exact “dose” or amount of time or “snacks” per day necessary to elicit meaningful muscular changes is unknown, however, the utility of such an intervention should not be overlooked to break up sedentary time and promote muscular fitness. Additionally, these snacks could provide benefits beyond physical health, as Wick et al. report teachers noted improved psychosocial behavior after these exercise breaks, especially in the younger children (≤4 years) (115). The ease of administration in conjunction with the physical and psychological benefits of “exercise snacks” warrants significant attention in future PA and early childhood research.

### 5.3 Bone health

Jumping is known to improve both hip and lumbar bone mass in prepubertal children (118), and exercise interventions for bone health in premenstrual girls are more effective than post menarche (119). Eight months of regular jumping in place for only 10 minutes, twice weekly during the school day can improve bone mass in older children as well (120). Even less of a stimulus can have a significant impact, as McKay et al. found that only 3 minutes/day of counter jump movements had a positive impact on bone health in elementary school aged children (121).

### 5.4 Current limitations in preschool data

Despite more research on PA among preschoolers, there have been well-documented limitations (81, 82, 98, 122) though sex differences may play a significant role in why previous work has been inconclusive with regards to PA and health outcomes. Some have found certain interventions effective only for improving PA in preschool girls (111, 123) while others have found a greater impact on boys (124, 125). These significant differences when stratified by sex suggest that the previous trials which saw no intervention effect, may have in fact been “washed out” by this variable and unable to mediate the confounder due to small sample sizes (107). In addition to sex, other influential factors to consider include socioeconomic status, as a recent large cohort analysis found associations with socioeconomic status and measures of musculoskeletal strength in preschool children (126).

## 6 Other considerations for implementation of PA interventions in infancy through early childhood

It is important to acknowledge that there are other limitations throughout infancy, toddlerhood and early childhood that are not discussed within this text, but highlighted in a recent systematic review (127). Within this section, however, we will highlight several factors that should be considered. Temperament, an early form of personality, is a behavioral style that is easily assessed in childhood as an encompassment of reaction to food, soothability, attention span, activity, sociability and emotionality (128). Even when controlling for other factors, childhood temperament is a robust predictor of adult BMI and influence choice in behaviors such as intensity of PA (129). In fact, Buss et al. found that several interpersonal attributes and personality descriptors were related to objectively measured PA levels in preschool children (99), although activity data collected during the school day has been shown to have no correlation with temperament in preschool children (130). Other individual-level factors to consider include sex (60), sleep (131), child interests (132), socioeconomic status (73), genetics (133), or epigenetics (134). Continuing research in the field should consider these factors as a means of identifying strategies to promote individualized and targeted behavior changes in infants, toddlers, and early childhood.

## 7 Conclusion: practical recommendations for researchers and providers

The childhood obesity epidemic requires the scientific community to better understand and address factors that mitigate weight gain, including the role of PA. Although infancy and the toddler years present unique and effective times to promote healthy habits, most interventions and guidelines for PA have focused on school age children and adolescents, with only modest success. Within this narrative review, we have highlighted the paucity of PA trials in early childhood. As a result, there is limited evidence to serve as the basis for PA guidelines for children <5 years old. Recognizing that the issue of obesity is extremely complex, we have proposed novel and modified interventions to promote improved health outcomes necessary for short-term motor development and health across the life course. Currently, no specific or easily accessible infant through early childhood PA recommendations exist for healthcare providers. This leaves them poorly equipped to promote PA in the office during a period in the child’s life where there are frequent provider/patient contacts and improved ability to promote behavior change. With this paper, we hope to prompt more research designed to develop and test innovative early childhood interventions that will equip providers and parents with effective approaches to combat childhood obesity and promote long-term health that persists into adulthood. Novel interventions at the individual and population level will allow for development of specific and applicable recommendations that healthcare providers can feel confident in prescribing to patients and their parents. Targeting PA during infancy through early childhood provides a distinctive lens through which researchers and clinicians in the world of pediatrics can work together to promote interventions and policies that will ensure healthy future generations of children and adults.

## Author contributions

NS, IP, and RP contributed to conception of the paper. NS and IP wrote the first draft of the manuscript. All authors contributed to manuscript revision, read, and approved the submitted version.
